# 8-Hy­droxy-5,7-dimethyl­quinolin-1-ium hydrogen sulfate

**DOI:** 10.1107/S1600536812049483

**Published:** 2012-12-08

**Authors:** Kaliyaperumal Thanigaimani, Nuridayanti Che Khalib, Suhana Arshad, Ibrahim Abdul Razak

**Affiliations:** a School of Physics, Universiti Sains Malaysia, 11800 USM, Penang, Malaysia

## Abstract

The quinoline ring system of the title salt, C_11_H_12_NO^+^·HSO_4_^−^, is essentially planar, with a maximum deviation of 0.054 (2) Å for all non H atoms. In the crystal, the cations and anions are linked *via* N—H⋯O, O—H⋯O and weak C—H⋯O hydrogen bonds, and are stacked respectively in columns along the *a* axis. π–π stacking inter­actions, with centroid–centroid distances of 3.5473 (12) and 3.6926 (12) Å, are also observed. The crystal studied was an inversion twin with refined components of 0.43 (7):0.57 (7).

## Related literature

For background to and the biological activity of quinoline derivatives, see: Sasaki *et al.* (1998 ▶); Reux *et al.* (2009 ▶); Morimoto *et al.* (1991 ▶); Markees *et al.* (1970 ▶). For related structures, see: Loh *et al.* (2010*a* ▶,*b* ▶). For bond-length data, see: Allen *et al.* (1987 ▶). For the stability of the temperature controller used for the data collection, see: Cosier & Glazer (1986 ▶).
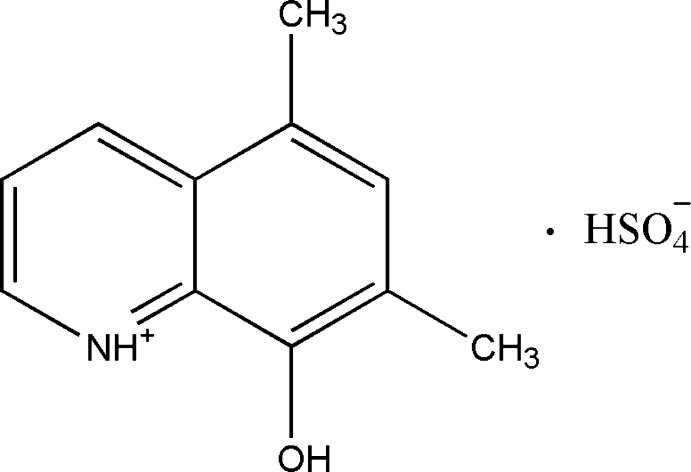


## Experimental

### 

#### Crystal data

C_11_H_12_NO^+^·HSO_4_^−^
*M* *_r_* = 271.28
Orthorhombic, 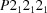

*a* = 6.6750 (9) Å
*b* = 11.6952 (14) Å
*c* = 14.7283 (18) Å
*V* = 1149.8 (3) Å^3^
*Z* = 4
Mo *K*α radiation
μ = 0.30 mm^−1^
*T* = 100 K
0.41 × 0.17 × 0.15 mm

#### Data collection

Bruker APEXII DUO CCD area-detector diffractometer
Absorption correction: multi-scan (*SADABS*; Bruker, 2009 ▶) *T*_min_ = 0.889, *T*_max_ = 0.956
9735 measured reflections
3341 independent reflections
3142 reflections with *I* > 2σ(*I*)
*R*_int_ = 0.040

#### Refinement

*R*[*F* ^2^ > 2σ(*F* ^2^)] = 0.039
*wR*(*F* ^2^) = 0.103
*S* = 1.05
3341 reflections
178 parameters
H atoms treated by a mixture of independent and constrained refinement
Δρ_max_ = 0.84 e Å^−3^
Δρ_min_ = −0.42 e Å^−3^
Absolute structure: Flack (1983 ▶), 1410 Friedel pairs
Flack parameter: 0.43 (7)

### 

Data collection: *APEX2* (Bruker, 2009 ▶); cell refinement: *SAINT* (Bruker, 2009 ▶); data reduction: *SAINT* (Bruker, 2009 ▶); program(s) used to solve structure: *SHELXTL* (Sheldrick, 2008 ▶); program(s) used to refine structure: *SHELXTL* (Sheldrick, 2008 ▶); molecular graphics: *SHELXTL* (Sheldrick, 2008 ▶); software used to prepare material for publication: *SHELXTL* (Sheldrick, 2008 ▶) and *PLATON* (Spek, 2009 ▶).

## Supplementary Material

Crystal structure: contains datablock(s) global, I. DOI: 10.1107/S1600536812049483/is5225sup1.cif

Structure factors: contains datablock(s) I. DOI: 10.1107/S1600536812049483/is5225Isup2.hkl

Supplementary material file. DOI: 10.1107/S1600536812049483/is5225Isup3.cml

Additional supplementary materials:  crystallographic information; 3D view; checkCIF report

## Figures and Tables

**Table 1:** Hydrogen-bond geometry (Å, °)

*D*—H⋯*A*	*D*—H	H⋯*A*	*D*⋯*A*	*D*—H⋯*A*
N1—H1*N*1⋯O3^i^	0.91 (2)	1.90 (2)	2.7753 (17)	161 (2)
O5—H1*O*5⋯O2^ii^	0.79 (3)	1.91 (3)	2.698 (2)	172 (2)
O1—H1*O*1⋯O4^i^	0.97 (4)	1.64 (4)	2.601 (2)	172 (3)
C3—H3*A*⋯O5^iii^	0.95	2.46	3.3448 (19)	154
C11—H11*C*⋯O3^ii^	0.98	2.50	3.445 (3)	161

Symmetry codes: (i) 
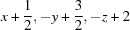
; (ii) 
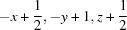
; (iii) 
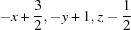
.

**Fig. 1.**
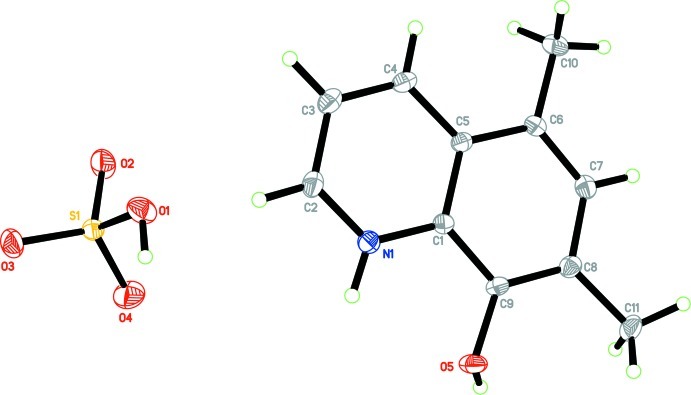
The molecular structure of the title compound with atom labels with 50% probability displacement ellipsoids.

**Fig. 2.**
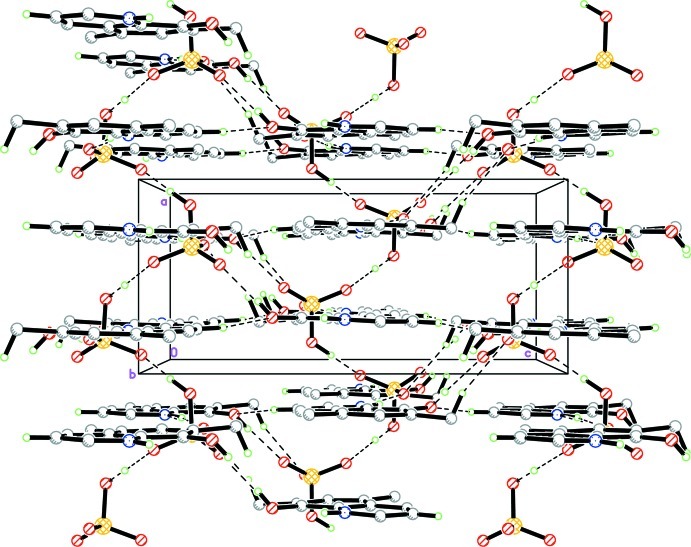
The crystal packing of the title compound. The H atoms not involved in the intermolecular interactions (dashed lines) have been omitted for clarity.

## supplementary crystallographic information

### Comment

Recently, hydrogen-bonding patterns involving quinoline and its derivatives with organic acid have been investigated (Loh *et al.*, 2010*a*,*b*). Syntheses of the quinoline derivatives were discussed earlier (Sasaki *et al.*, 1998; Reux *et al.*, 2009). Quinolines and their derivatives are very important compounds because of their wide occurrence in natural products (Morimoto *et al.*, 1991) and biologically active compounds (Markees *et al.*, 1970). Herein we report the synthesis of 8-hydroxy-5,7-dimethylquinolin-1-ium hydrogen sulfate.

The asymmetric unit of the title compound (Fig. 1) consists of one 8-hydroxy-5,7-dimethylquinolin-1-ium cation and one hydrogen sulfate anion. One proton is transferred from the hydroxyl group of sulfuric acid to the atom N1 of 8-hydroxy-5,7-dimethylquinoline during the crystallization, resulting in the formation of salt. The quinoline ring system (C1–C9/N1) is essentially planar with a maximum deviation of 0.054 (2) Å at atom C8. The bond lengths (Allen *et al.*, 1987) and angles are normal.

In the crystal packing (Fig. 2), the cations are linked by the anions *via* intermolecular N1—H1N1···O3^i^, O5—H1O5···O2^ii^, O1—H1O1···O4^i^, C3—H3A···O5^iii^ and C11—H11C···O3^ii^ hydrogen bonds (symmetry codes in Table 1) into a three-dimensional network. Furthermore, the crystal structure is stabilized by the following π–π interactions: (*a*) between pyridine (N1/C1–C5, centroid *Cg*1) and benzene (C1/C5–C9, centroid *Cg*2) rings *Cg*1···*Cg*2 (1/2 + *x*, 1/2 - *y*, 2 - *z*) 3.5473 (12) Å and (*b*) between benzene rings (C1/C5–C9, centroid *Cg*2) *Cg*2···*Cg*2 (-1/2 + *x*, 1/2 - *y*, 2 - *z*) 3.6926 (12) Å. The crystal studied was an inversion twin, with a ratio of the twin components of 0.43 (7):0.57 (7).

### Experimental

A few drops of sulfuric acid were added to a hot methanol solution (20 ml) of 8-hydroxy-5,7-dimethylquinoline (36 mg, Aldrich) which had been warmed over a heating magnetic stirrer hotplate for a few minutes. The resulting solution was allowed to cool slowly at room temperature and crystals of the title compound (I) appeared after a few days.

### Refinement

O- and N-bound H atoms were located in a difference Fourier map and refined freely [refined distances: O—H = 0.97 (4) and 0.79 (3) Å, N—H = 0.91 (2) Å]. The remaining hydrogen atoms were positioned geometrically (C—H = 0.95–0.98 Å) and were refined using a riding model, with *U*_iso_(H) = 1.2 *U*_eq_(C) or 1.5*U*_eq_(methyl C). A rotating-group model was used for the methyl group. The crystal studied was an inversion twin, with a ratio of the twin components of 0.43 (7):0.57 (7). The Hooft y parameter was 0.48 (4).

### Crystal data

**Table:**

C_11_H_12_NO^+^·HSO_4_^−^	*F*(000) = 568
*M**_r_* = 271.28	*D*_x_ = 1.567 Mg m^−^^3^
Orthorhombic, *P*2_1_2_1_2_1_	Mo *K*α radiation, λ = 0.71073 Å
Hall symbol: P 2ac 2ab	Cell parameters from 6153 reflections
*a* = 6.6750 (9) Å	θ = 2.8–29.9°
*b* = 11.6952 (14) Å	µ = 0.30 mm^−^^1^
*c* = 14.7283 (18) Å	*T* = 100 K
*V* = 1149.8 (3) Å^3^	Block, yellow
*Z* = 4	0.41 × 0.17 × 0.15 mm

### Data collection

**Table:**

Bruker APEXII DUO CCD area-detector diffractometer	3341 independent reflections
Radiation source: fine-focus sealed tube	3142 reflections with *I* > 2σ(*I*)
Graphite monochromator	*R*_int_ = 0.040
φ and ω scans	θ_max_ = 30.0°, θ_min_ = 2.8°
Absorption correction: multi-scan (*SADABS*; Bruker, 2009)	*h* = −9→9
*T*_min_ = 0.889, *T*_max_ = 0.956	*k* = −16→16
9735 measured reflections	*l* = −20→20

### Refinement

**Table:**

Refinement on *F*^2^	Secondary atom site location: difference Fourier map
Least-squares matrix: full	Hydrogen site location: inferred from neighbouring sites
*R*[*F*^2^ > 2σ(*F*^2^)] = 0.039	H atoms treated by a mixture of independent and constrained refinement
*wR*(*F*^2^) = 0.103	*w* = 1/[σ^2^(*F*_o_^2^) + (0.0699*P*)^2^ + 0.0691*P*] where *P* = (*F*_o_^2^ + 2*F*_c_^2^)/3
*S* = 1.05	(Δ/σ)_max_ = 0.001
3341 reflections	Δρ_max_ = 0.84 e Å^−^^3^
178 parameters	Δρ_min_ = −0.42 e Å^−^^3^
0 restraints	Absolute structure: Flack (1983), 1410 Friedel pairs
Primary atom site location: structure-invariant direct methods	Flack parameter: 0.43 (7)

### Special details

**Table:**

Experimental. The crystal was placed in the cold stream of an Oxford Cryosystems Cobra open-flow nitrogen cryostat (Cosier & Glazer, 1986) operating at 100.0 (1) K.
Geometry. All e.s.d.'s (except the e.s.d. in the dihedral angle between two l.s. planes) are estimated using the full covariance matrix. The cell e.s.d.'s are taken into account individually in the estimation of e.s.d.'s in distances, angles and torsion angles; correlations between e.s.d.'s in cell parameters are only used when they are defined by crystal symmetry. An approximate (isotropic) treatment of cell e.s.d.'s is used for estimating e.s.d.'s involving l.s. planes.
Refinement. Refinement of *F*^2^ against ALL reflections. The weighted *R*-factor *wR* and goodness of fit *S* are based on *F*^2^, conventional *R*-factors *R* are based on *F*, with *F* set to zero for negative *F*^2^. The threshold expression of *F*^2^ > σ(*F*^2^) is used only for calculating *R*-factors(gt) *etc*. and is not relevant to the choice of reflections for refinement. *R*-factors based on *F*^2^ are statistically about twice as large as those based on *F*, and *R*- factors based on ALL data will be even larger.

### Fractional atomic coordinates and isotropic or equivalent isotropic displacement parameters (Å^2^)

**Table:**

	*x*	*y*	*z*	*U*_iso_*/*U*_eq_	
S1	0.15917 (7)	0.73295 (3)	0.89075 (3)	0.01680 (11)	
O1	0.3784 (2)	0.68512 (11)	0.89220 (9)	0.0235 (3)	
O2	0.0640 (3)	0.66640 (10)	0.82023 (10)	0.0284 (3)	
O3	0.1707 (3)	0.85435 (10)	0.87008 (9)	0.0270 (3)	
O4	0.0779 (3)	0.70976 (12)	0.98112 (10)	0.0307 (3)	
O5	0.7154 (2)	0.37726 (9)	1.19245 (7)	0.0180 (3)	
N1	0.7224 (2)	0.45539 (10)	1.01976 (9)	0.0145 (3)	
C1	0.7217 (3)	0.33937 (12)	1.03458 (9)	0.0126 (3)	
C2	0.7309 (3)	0.50147 (13)	0.93688 (10)	0.0165 (3)	
H2A	0.7349	0.5822	0.9302	0.020*	
C3	0.7339 (3)	0.43178 (14)	0.85994 (10)	0.0175 (3)	
H3A	0.7394	0.4646	0.8010	0.021*	
C4	0.7288 (3)	0.31450 (14)	0.87066 (9)	0.0158 (3)	
H4A	0.7283	0.2665	0.8186	0.019*	
C5	0.7243 (3)	0.26499 (13)	0.95828 (9)	0.0128 (3)	
C6	0.7217 (3)	0.14410 (12)	0.97396 (10)	0.0138 (3)	
C7	0.7275 (3)	0.10657 (13)	1.06266 (10)	0.0156 (3)	
H7A	0.7291	0.0265	1.0733	0.019*	
C8	0.7312 (3)	0.18055 (14)	1.13898 (10)	0.0151 (3)	
C9	0.7197 (3)	0.29722 (12)	1.12470 (9)	0.0137 (3)	
C10	0.7169 (3)	0.06073 (13)	0.89626 (11)	0.0189 (3)	
H10A	0.7094	−0.0174	0.9201	0.028*	
H10B	0.8388	0.0692	0.8598	0.028*	
H10C	0.5994	0.0760	0.8583	0.028*	
C11	0.7464 (3)	0.13120 (14)	1.23285 (11)	0.0203 (4)	
H11A	0.8315	0.1804	1.2704	0.030*	
H11B	0.8052	0.0545	1.2296	0.030*	
H11C	0.6125	0.1264	1.2598	0.030*	
H1N1	0.713 (4)	0.5064 (18)	1.0663 (15)	0.015 (5)*	
H1O5	0.630 (5)	0.359 (2)	1.227 (2)	0.038 (8)*	
H1O1	0.454 (6)	0.718 (3)	0.942 (2)	0.060 (10)*	

### Atomic displacement parameters (Å^2^)

**Table:**

	*U*^11^	*U*^22^	*U*^33^	*U*^12^	*U*^13^	*U*^23^
S1	0.0199 (2)	0.01535 (16)	0.01516 (16)	0.00047 (15)	−0.00309 (16)	−0.00102 (12)
O1	0.0210 (7)	0.0244 (5)	0.0250 (6)	0.0039 (5)	0.0003 (6)	−0.0053 (5)
O2	0.0380 (9)	0.0183 (5)	0.0289 (6)	−0.0014 (6)	−0.0167 (7)	−0.0033 (5)
O3	0.0395 (9)	0.0146 (5)	0.0268 (6)	−0.0008 (6)	−0.0120 (6)	−0.0010 (4)
O4	0.0316 (8)	0.0373 (7)	0.0232 (6)	0.0046 (7)	0.0116 (6)	0.0026 (5)
O5	0.0252 (7)	0.0174 (5)	0.0113 (4)	−0.0031 (5)	0.0034 (5)	−0.0027 (4)
N1	0.0161 (7)	0.0135 (5)	0.0139 (5)	0.0003 (5)	0.0017 (5)	0.0007 (4)
C1	0.0130 (7)	0.0129 (6)	0.0120 (6)	0.0003 (6)	0.0007 (6)	0.0000 (5)
C2	0.0174 (8)	0.0151 (6)	0.0170 (6)	0.0011 (6)	0.0006 (7)	0.0044 (5)
C3	0.0179 (9)	0.0205 (7)	0.0142 (6)	−0.0002 (7)	0.0005 (7)	0.0039 (5)
C4	0.0169 (8)	0.0193 (6)	0.0112 (6)	−0.0002 (6)	0.0003 (6)	0.0004 (5)
C5	0.0125 (7)	0.0153 (6)	0.0107 (5)	0.0004 (6)	0.0000 (5)	−0.0002 (5)
C6	0.0136 (7)	0.0133 (6)	0.0146 (6)	0.0003 (6)	0.0005 (6)	−0.0020 (5)
C7	0.0151 (8)	0.0141 (6)	0.0177 (6)	0.0007 (6)	0.0018 (6)	0.0012 (5)
C8	0.0139 (8)	0.0180 (6)	0.0134 (6)	0.0006 (6)	0.0011 (6)	0.0016 (5)
C9	0.0159 (7)	0.0156 (6)	0.0098 (6)	−0.0014 (6)	−0.0005 (6)	−0.0008 (5)
C10	0.0219 (9)	0.0172 (6)	0.0176 (6)	−0.0010 (6)	0.0010 (7)	−0.0053 (5)
C11	0.0240 (10)	0.0219 (7)	0.0149 (6)	0.0038 (7)	0.0012 (7)	0.0067 (5)

### Geometric parameters (Å, º)

**Table:**

S1—O2	1.4451 (13)	C4—C5	1.4148 (18)
S1—O3	1.4542 (12)	C4—H4A	0.9500
S1—O4	1.4626 (14)	C5—C6	1.433 (2)
S1—O1	1.5668 (15)	C6—C7	1.379 (2)
O1—H1O1	0.97 (4)	C6—C10	1.504 (2)
O5—C9	1.3685 (17)	C7—C8	1.419 (2)
O5—H1O5	0.79 (3)	C7—H7A	0.9500
N1—C2	1.3355 (18)	C8—C9	1.383 (2)
N1—C1	1.3743 (17)	C8—C11	1.502 (2)
N1—H1N1	0.91 (2)	C10—H10A	0.9800
C1—C9	1.4160 (18)	C10—H10B	0.9800
C1—C5	1.4212 (19)	C10—H10C	0.9800
C2—C3	1.396 (2)	C11—H11A	0.9800
C2—H2A	0.9500	C11—H11B	0.9800
C3—C4	1.381 (2)	C11—H11C	0.9800
C3—H3A	0.9500		
			
O2—S1—O3	113.50 (8)	C1—C5—C6	118.45 (12)
O2—S1—O4	113.03 (10)	C7—C6—C5	117.81 (13)
O3—S1—O4	113.04 (8)	C7—C6—C10	121.01 (13)
O2—S1—O1	103.19 (8)	C5—C6—C10	121.17 (13)
O3—S1—O1	107.56 (9)	C6—C7—C8	123.86 (14)
O4—S1—O1	105.54 (9)	C6—C7—H7A	118.1
S1—O1—H1O1	111 (2)	C8—C7—H7A	118.1
C9—O5—H1O5	108 (2)	C9—C8—C7	118.70 (13)
C2—N1—C1	122.94 (13)	C9—C8—C11	121.54 (14)
C2—N1—H1N1	115.2 (14)	C7—C8—C11	119.75 (14)
C1—N1—H1N1	121.8 (14)	O5—C9—C8	124.42 (13)
N1—C1—C9	119.52 (13)	O5—C9—C1	116.46 (13)
N1—C1—C5	118.60 (13)	C8—C9—C1	119.05 (13)
C9—C1—C5	121.89 (13)	C6—C10—H10A	109.5
N1—C2—C3	120.46 (14)	C6—C10—H10B	109.5
N1—C2—H2A	119.8	H10A—C10—H10B	109.5
C3—C2—H2A	119.8	C6—C10—H10C	109.5
C4—C3—C2	119.12 (14)	H10A—C10—H10C	109.5
C4—C3—H3A	120.4	H10B—C10—H10C	109.5
C2—C3—H3A	120.4	C8—C11—H11A	109.5
C3—C4—C5	120.74 (13)	C8—C11—H11B	109.5
C3—C4—H4A	119.6	H11A—C11—H11B	109.5
C5—C4—H4A	119.6	C8—C11—H11C	109.5
C4—C5—C1	118.11 (13)	H11A—C11—H11C	109.5
C4—C5—C6	123.44 (13)	H11B—C11—H11C	109.5
			
C2—N1—C1—C9	177.73 (17)	C1—C5—C6—C10	177.83 (16)
C2—N1—C1—C5	−1.9 (3)	C5—C6—C7—C8	1.5 (3)
C1—N1—C2—C3	1.8 (3)	C10—C6—C7—C8	−179.68 (18)
N1—C2—C3—C4	−0.3 (3)	C6—C7—C8—C9	2.9 (3)
C2—C3—C4—C5	−1.2 (3)	C6—C7—C8—C11	−177.37 (18)
C3—C4—C5—C1	1.1 (3)	C7—C8—C9—O5	177.81 (17)
C3—C4—C5—C6	−179.12 (17)	C11—C8—C9—O5	−1.9 (3)
N1—C1—C5—C4	0.4 (2)	C7—C8—C9—C1	−5.3 (3)
C9—C1—C5—C4	−179.20 (17)	C11—C8—C9—C1	175.01 (17)
N1—C1—C5—C6	−179.41 (16)	N1—C1—C9—O5	1.0 (3)
C9—C1—C5—C6	1.0 (3)	C5—C1—C9—O5	−179.39 (16)
C4—C5—C6—C7	176.82 (17)	N1—C1—C9—C8	−176.17 (17)
C1—C5—C6—C7	−3.4 (3)	C5—C1—C9—C8	3.4 (3)
C4—C5—C6—C10	−2.0 (3)		

### Hydrogen-bond geometry (Å, º)

**Table:**

*D*—H···*A*	*D*—H	H···*A*	*D*···*A*	*D*—H···*A*
N1—H1*N*1···O3^i^	0.91 (2)	1.90 (2)	2.7753 (17)	161 (2)
O5—H1*O*5···O2^ii^	0.79 (3)	1.91 (3)	2.698 (2)	172 (2)
O1—H1*O*1···O4^i^	0.97 (4)	1.64 (4)	2.601 (2)	172 (3)
C3—H3*A*···O5^iii^	0.95	2.46	3.3448 (19)	154
C11—H11*C*···O3^ii^	0.98	2.50	3.445 (3)	161

Symmetry codes: (i) *x*+1/2, −*y*+3/2, −*z*+2; (ii) −*x*+1/2, −*y*+1, *z*+1/2; (iii) −*x*+3/2, −*y*+1, *z*−1/2.
